# Stress granules: potential therapeutic targets for infectious and inflammatory diseases

**DOI:** 10.3389/fimmu.2023.1145346

**Published:** 2023-05-02

**Authors:** Wenyuan Li, Yao Wang

**Affiliations:** ^1^ Department of Anesthesiology, Renmin Hospital of Wuhan University, Wuhan, Hubei, China; ^2^ Department of Infectious Diseases, Renmin Hospital of Wuhan University, Wuhan, Hubei, China

**Keywords:** stress granules, infection, inflammation, innate immunity, therapeutic targets

## Abstract

Eukaryotic cells are stimulated by external pressure such as that derived from heat shock, oxidative stress, nutrient deficiencies, or infections, which induce the formation of stress granules (SGs) that facilitates cellular adaptation to environmental pressures. As aggregated products of the translation initiation complex in the cytoplasm, SGs play important roles in cell gene expression and homeostasis. Infection induces SGs formation. Specifically, a pathogen that invades a host cell leverages the host cell translation machinery to complete the pathogen life cycle. In response, the host cell suspends translation, which leads to SGs formation, to resist pathogen invasion. This article reviews the production and function of SGs, the interaction between SGs and pathogens, and the relationship between SGs and pathogen-induced innate immunity to provide directions for further research into anti-infection and anti-inflammatory disease strategies.

## 1 Introduction

When cells are subjected to various degrees of damage, such as that induced by viral or bacterial infection, heat shock, oxidative stress, or nutrient starvation, membrane-less RNA/protein molecular condensates, known as stress granules (SGs), are formed in the cytoplasm (1). The generation of SGs is generally believed to be caused by abrogated translation that is induced to prevent abnormal protein synthesis, with the transcription and translation of translation-stalled nucleoproteins resuming in the absence of external stimuli or with SGs being captured during processing body (PB) disassembly (2). SGs are dynamic membrane-free cytoplasmic structures that are rapidly modified in response to changes in the external environment (3). SGs in the cytoplasm are complexes comprising protein and RNA, and the SGs composition varies according to different conditions (4).

Studies have shown that SGs are closely related to viral infections (5), genetic diseases (6), tumors (7) and other diseases. Because SGs are involved in innate immune regulatory processes, most of the studies on SGs have been focused on the field of virology. Viruses are characterized by their ability to hinder the growth of host cells in a way that is conducive to viral replication and spread, and many virus-infected cells show an increased SGs formation rate and blocked protein translation. Since a virus relies on the cellular translation mechanism for protein synthesis, SGs can hinder the replication of a virus in a cell while affecting the expression of cellular proteins. To escape host cell resistance, viruses have evolved various strategies to disrupt SGs formation and promote their own replication (8). The formation of SGs in most virus-infected cells is caused by the phosphorylation of eukaryotic translation initiation factor (TIF) 2α (eIF2α) through the activation of protein kinase R (PKR). SGs, as players downstream of the cellular stress response, may play a role in viral replication and may be promoters of cellular innate immune responses to viral infection.

An RNA virus or a DNA virus can induce or regulate the production of SGs in host cells during infection. Viruses play different regulatory roles on SGs formation throughout the replication cycle. Moreover, SGs act on viruses. Although research on the relationship between bacterial infection and SGs is in its infancy, bacteria are clearly closely related to SGs formation and change the level of intracellular p-eIF2α (9). Moreover, similar to cellular responses and viral infection, SGs and cellular inflammatory responses are related. For example, recent reports have shown that SGs attenuated macrophage-mediated cell inflammatory damage (10) and further regulated cell survival and pyroptosis by inhibiting inflammasome activation (10). In contrast, certain proinflammatory factors, such as IFN-γ and TNF-α, promote the formation of SGs through the phosphorylation of eIF2α (11). Despite reports indicating correlations between infective (viruses and bacteria) inflammatory factors and SGs, the molecular mechanisms underlying SGs formation remain unclear. Therefore, the correlations between infective and inflammatory factors and SGs deserves further exploration. This article reviews recent reports on the relationship among infective and inflammatory factors and SGs to identify ideas for finding new therapeutic targets for infection and inflammation.

## 2 SGs formation

### 2.1 SGs components

**Figure 1:**
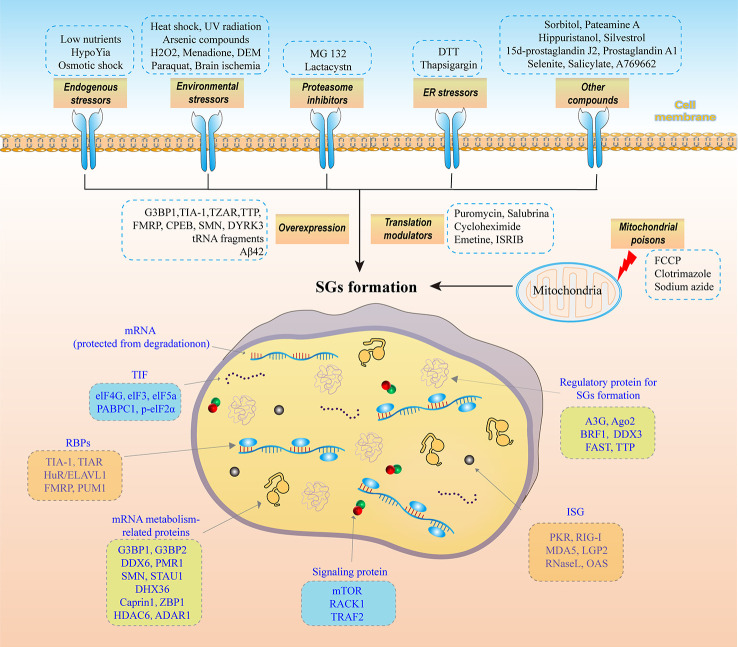
The main components of SGs and the factors that induce SGs formation. SGs comprise the following 7 components: (1) mRNAs that are protected from degradation. (2) TIFs targeting mRNAs, such as eIF4E, elF3, PABPC1, p-elF2α and elF5a; (3) RNA-binding proteins (RBPs) that regulate translation and protect mRNA stability, such as TIA-1, TIAR, HuR/ELAVL1, FMRP and PUM1; (4) mRNA metabolism-related proteins, such as G3BP1, G3BP2, DDX6, PMR1, SMN, STAU1, DHX36, caprin-1, ZBP1, HDAC6 and ADAR1; (5) signaling proteins, such as mTOR, RACK1 and TRAF; (6) expression products of interferon-stimulated genes (ISGs), such as PKR, RIG-I, MDA5 and LGP2, RNase L and OAS; and (7) regulatory proteins in SGs formation, such as APOBEC3G, Ago2, BRF1, DDX3, FAST and TTP. Eight factors induce SGs formation: (1) endogenous stressors, such as low-level nutrients, hypoxia, and osmotic shock; (2) environmental stressors, such as heat shock, UV radiation, arsenic compounds, H_2_O_2_, menadione, diethyl maleate (DEM), paraquat, and brain ischemia; (3) overexpressed G3BP1, TIA-1, TZAR, TTP, FMRP, CPEB, SMN, DYRK3, tRNA fragments, and Aβ42; (4) translation modulators, such as puromycin, salubrinal, cycloheximide, emetine, and ISRIB; (5) proteasome inhibitors, such as MG 132 and lactacystin; (6) ER stressors, such as DTT and thapsigargin; (7) mitochondrial poisons, such as FCCP, clotrimazole, and sodium azide; and (8) other compounds, such as sorbitol, pateamine A, hippuristanol, silvestrol, 15d-prostaglandin J2 (15d-PGJ2); prostaglandin A1, selenite, salicylate, and A769662.

SGs are membraneless organelles produced by cells in response to stress-inducing external environmental stimuli (such as inflammatory stimuli, heat shock, oxidative stress, ischemia or viral infection) and temporarily abrogate the translation of most mRNAs. These untranslated mRNAs and related RNA-binding proteins (RBPs) form dynamic SGs in the cytoplasm. Studies have shown that the main components, which are shown in **Figure 1**; specifically, SGs comprise the following 7 components: (1) translation-blocked mRNAs that are protected from degradation (12); (2) mRNA-targeted TIFs, such as eukaryotic initiation factor 4E (eIF4E), elF3, poly(A)-binding protein 1 (PABPC1), phosphorylated elF2α (p-elF2α) and elF5a (13); (3) RBPs that regulate translation and protect mRNA stability, such as T-cell-restricted intracellular antigen-1 (TIA-1), TIA-related protein (TIAR), human antigen R/Elav-like RNA binding protein 1 (HuR/ELAVL1), fragment X intellectual disability protein (FMRP) and pumilio RNA-binding family member 1 (PUM1) (13); (4) mRNA metabolism-related proteins, such as Ras GTPase-activating protein-binding protein 1 (G3BP1), G3BP2, DEAD-box RNA helicase 6 (DDX6 or Rck/p54), plasma membrane-related Ca^2+^-ATPase 1 (PMR1), survival motor neuron protein (SMN), staufen1 (STAU1), DEAH (Asp-Glu-Ala-His) box polypeptide 36 (DHX36), cytoplasmic activation/proliferation-associated protein-1 (caprin-1), Z-DNA-binding protein 1 (ZBP1), histone deacetylase 6 (HDAC6) and adenosine deaminase acting on RNA1 (ADAR1) (14); (5) signaling proteins, such as mechanistic target of rapamycin (mTOR), receptor of activated protein kinase C1 (RACK1) and TNF receptor-associated factor 2 (TRAF2) (15); (6) expression products of interferon-stimulated genes (ISGs), such as PKR, RNA-sensing RIG-I-like receptor (RIG-I), melanoma differentiation-associated gene 5 (MDA5) and laboratory of genetics and physiology 2 (LGP2), ribonuclease L (RNase L) and oligoadenylate synthetase (OAS) (16); and (7) regulatory proteins involved in SGs formation, such as apolipoprotein B mRNA-editing catalytic polypeptide enzyme-like 3G (APOBEC3G or A3G), argonaute 2 (Ago2), B-related factor 1 (BRF1), DDX3, Fas-activated serine/threonine phosphoprotein (FAST) and tristetraprolin (TTP) (17).

Notably, G3BP1 is a phosphorylation-dependent endonuclease and a core protein that mediates SGs assembly. G3BP1 consists of an N-terminal nuclear transport factor 2 (NTF2) domain, a C-terminal receptor-binding domain (RBD) and an intermediate intrinsically disordered region (IDR) (18). The formation of SGs begins with the establishment of a core SGs network, which includes the G3BP1 protein, with interwoven protein-protein, protein-RNA, and RNA-RNA interacting pairs. In a mutually synergistic process, the G3BP1 complex induces phase separation, which leads to SGs assembly (19). Studies have shown that SGs assembly can be completely inhibited only after both G3BP1 and G3BP2 (G3BP2 and G3BP1 are highly similar) are knocked out (19). However, knocking out G3BP1, G3BP2, DDX3X, Caprin1 or another important SGs component alone does not completely inhibit SGs assembly. In addition, a G3BP1-mutant protein lacking the NTF2 or RBD domain failed to mediate SGs assembly, while G3BP1 with the both dual deletion of IDR1 and IDR2 mediated the SGs assembly process (20).

### 2.2 The driving force of SGs assembly: liquid–liquid phase separation

Hyman et al. first introduced the physicochemical concept of LLPS into biological research with the study of P granules in 2009 (21). Cell differentiation fate is determined through the asymmetric distribution of P granules as mediated by LLPS. LLPS in cells is initiated by the collision among different proteins and nucleic acid molecules, ultimately leading to aggregates that form droplet-like structures through multivalent weak interactions and ultimately specific cellular compartments. In addition to P granules, intracellular membraneless organelles such as SGs and paraspeckles are formed *via* LLPS (22). LLPS explains how specific molecules in cells aggregate, and how these aggregates are maintained in a state with specific fluidity without the involvement of membrane structures and physiologically function in specific areas within cells. Hence, LLPS is considered to be the main driver of membraneless organelle formation (23).

However, the fundamental criteria underlying the assembly and structure of SGs differ from those of similar P granule structures. Ribosomal machinery is constantly moving along the RNA assembly line, isolating genetic information from the environment and producing proteins necessary for the cell to survive. When external pressure disrupts the assembly line, the RNA is stripped away from ribosomes and aggregated into SGs and P granules. SGs protein assembly requires a specific set of building blocks that require a certain number of chemical linkers that grab RNA strands to bring them together. This linker, called an RNA-binding domain, is encoded in a variety of proteins. However, these linkers show different affinities that determine their biological functions. G3BP1 is the most abundant SGs protein with the greatest network affinities. Specifically, G3BP1 preferentially interacts with other proteins that also bind RNA. The reason that SGs and P granules are found together is that their networks overlap, exhibiting glue-like adhesiveness (18). In addition, membrane organelles affect the formation and function of SGs through LLPS; for example, the endoplasmic reticulum (ER) directly contacts SGs through ribonucleoprotein (RNP) bodies and thus regulates LLPS-mediated separation of SGs components (24). The membranous structure of the ER provides a platform for phase separation and actively participates in LLPS regulation. Conversely, LLPS affects the organization, storage, and release of membranous organelles.

### 2.3 Mechanism of SGs formation *via* p-eIF2α-dependent and -independent pathways

**Figure 2:**
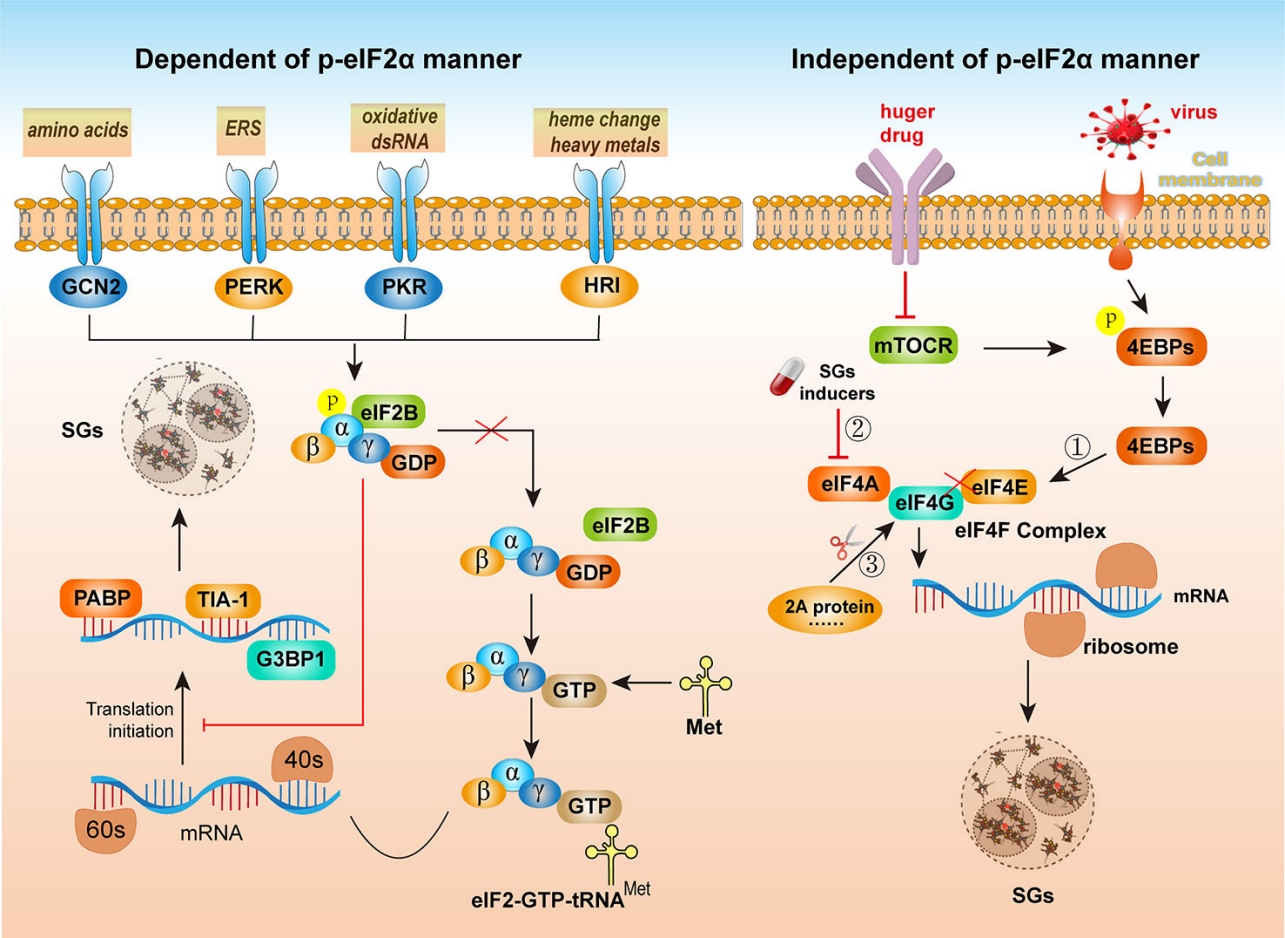
The formation of SGs is dependent or independent of eIF2α phosphorylation. (Left) *Via* different external stimuli, such as amino acids, ER stress (ERS), oxidative double-stranded RNA (dsRNA), and changes in heme heavy metal levels, four kinases, GCN2, PERK, PKR and HRI, can be activated. The α subunit of eIF2 is phosphorylated, preventing its separation from eIF2B. Subsequently, translation initiation is blocked. The mRNAs and certain proteins involved in the translation process aggregate to form SGs. Before recruiting the 60S ribosome to start translation, the eIF2-GTP-tRNA^Met^ trimer complex is recruited to the mRNA/eIF4F complex bound to the 40S ribosomal subunit, presenting messenger RNA linked to the translation initiation amino acid to the 40S ribosomal subunit. However, when eIF2 and eIF2B are separated, the ternary complex of eIF2-GTP-tRNA^Met^ is formed. The complex then binds to mRNA and 40S and 60S ribosomes and participates in translation initiation. Phosphorylated eIF2α interferes with the formation of the eIF2-GTP-tRNAMet complex, resulting in the retention of a large number of translation initiation complexes in the cytoplasm and ultimately induces SGs formation. (Left) The p-eIF2α-independent pathway formed by SGs requires disruption of the eIF4F complex to interfere with RNA helicase eIF4E activity and inhibit its interaction with eIF4G. Maintaining the structural and functional integrity of eIF4F is required for translation initiation and can induce SGs formation through the following three mechanisms: ① Inhibition of the interaction between eIF4E and eIF4G. The recruitment of eIF4F to mRNA may be regulated by mTOR. When cells are starved or damaged by drugs, mTOR is inactivated, resulting in the accumulation of unphosphorylated 4EBP in cells. Unphosphorylated 4EBP competes with eIF4F to bind eIF4E, preventing the formation of eIF4F. Therefore, inhibiting the initiation of translation results in a reduction in the number of polysomes, and translationally stopped preinitiation complexes (PICs) accumulate in the cell and are thus assembled into SGs; ② Inhibition of the interaction between eIF4A and eIF4G or inhibition of the RNA helicase activity of eIF4A. ③ Destruction of the eIF4G structure. For example, after CVB infection, the 2A protein cleaves eIF4G.

The evidence obtained thus far has suggested that the formation of SGs is achieved mainly by the phosphorylation of eIF2α and interference in the function of eIF4F, namely, *via* the p-eIF2α-dependent pathway and p-eIF2α-independent pathways (12). As shown on the left side of **Figure 2**, the phosphorylation of the α subunit of eIF2 is the most important initiator of SGs formation, as translation initiation is regulated by the phosphorylation of eIF2α. Four serine/threonine kinases can sense different types of stresses and phosphorylate the serine residue at position 51 of eIF2α: heme-regulated eIF2α kinase (HRI), general control nonderepressible 2 (GCN2), PKR and PKR-like ER kinase (PERK). GCN2 mainly senses the activation of intracellular stress caused by amino acid starvation (25). PERK is activated by endoplasmic reticulum stress (ERS) (26). PKR is a component of the interferon response mechanism and is activated by oxidative double-stranded RNA (dsRNA) in cells (27). HRI is activated after sensing changes in heme heavy metal levels, oxidative stress, thermal aggression, etc. (28).

eIF2B is an important protein involved in eukaryotic translation initiation. During translation initiation, eIF2-bound GDP (eIF2-GDP, the inactive state of eIF2) can be converted to eIF2-bound GTP (eIF2-GTP, the active state of eIF2). Therefore, when present in a sufficient amount, eIF2-GTP participates in translation initiation (29). When GCN2, PERK, PKR, and HRI kinases are activated, the α subunit of eIF2 is phosphorylated, preventing its separation from eIF2B. Subsequently, translation initiation is blocked, and mRNAs and certain proteins involved in the translation process aggregate to form SGs. In these cases, dsRNA formation or ERS induced by viral infection activates PKR and PERK, induces eIF2α phosphorylation, and leads to SGs formation. The formation of the eIF2-GTP-tRNA^Met^ trimer complex is also a key step in the cellular regulation of translation. Before recruiting the 60S ribosome to start translation, the eIF2-GTP-tRNA^Met^ trimer complex is recruited to the mRNA/eIF4F complex bound to the 40S ribosomal subunit, thereby presenting the messenger RNA linked to the translation initiation amino acid to 40S of the ribosomal subunit. However, when eIF2 and eIF2B are separated, a ternary complex with tRNA^Met^, eIF2-GTP-tRNA^Met^, is formed. This ternary complex binds to mRNA and the 40S and 60S ribosome subunits and thus participate in the translation initiation process. Phosphorylated eIF2α interferes with the formation of the eIF2-GTP-tRNA^Met^ complex, resulting in the retention of a large number of translation initiation complexes in the cytoplasm and ultimately inducing SGs formation (30). Therefore, when eIF2α is phosphorylated, the efficiency of the eIF2-GDP-to-EIF2-GTP conversion is inhibited, resulting in the inability of tRNAMet to bind to eIF2, inhibiting the formation of the eIF2-GTP-tRNAMet trimer complex and thereby inhibiting translation initiation. Because eIF2α phosphorylation does not affect translation elongation, ribosomes that have already begun the translation process are detached from mRNA, resulting in the polysome depolymerization, and cells accumulate a large number of 48S units lacking pretranslational eIF2, eIF5, or tRNA^Met^ and show no translation initiation function, called preinitiation complexes (PICs), which are assembled into SGs (31).

As shown in the right side of **Figure 2**, the eIF2α phosphorylation-independent pathway formation of SGs requires disruption of the eIF4F complex and interference with RNA helicase eIF4E activity to inhibit its interaction with eIF4G. During translation initiation, eIF4F is recruited by the vast majority of mRNAs in the initial step of translation. eIF4F is a heterotrimer consisting of eIF4A, eIF4E and eIF4G. eIF4A is an ATPase activity-dependent RNA helicase that opens the secondary structure of RNA and facilitates translation initiation complex scanning of RNA to recognize the AUG start codon. eIF4E binds to the cap structure at the 5’ end of mRNA, and the interaction between the eIF4E and mRNA mediates the recruitment of eIF4F *via* mRNA. The N-terminus of eIF4G interacts with eIF4E, and the C-terminus of eIF4G interacts with eIF4A, which forms a bridge to eIF4F. Maintaining the structural and functional integrity of eIF4F is required for translation initiation and can induce the formation of SGs through the following three mechanisms: (1) inhibition of the interaction between eIF4E and eIF4G. The recruitment of eIF4F to mRNA is regulated by mTOR, which is a serine/threonine kinase that controls cellular metabolism by regulating cellular protein synthesis (32). Under normal growth conditions, mTOR is activated. Phosphorylated eIF4E-binding proteins (4EBPs), however, cannot bind eIF4E. Therefore, eIF4F is formed, and translation proceeds normally. When cells are starved and damaged by drugs, mTOR is inactive. The accumulation of the unphosphorylated form of 4EBP in cells competes with eIF4F for binding to eIF4E, preventing the formation of eIF4F-mRNA complexes, thereby inhibiting the initiation of translation. This abrogated translation results in a reduction in the number of polysomes, and the PICs that accumulate in cells lacking key initiation factors are assembled into SGs. Similarly, tRNA-derived stress-induced RNAs (tiRNAs), produced by angiogenin-induced cleavage of mature messenger RNA, can dissociate eIF4G/eIF4A from eIF4E bound to the mRNA cap structure to generate PICs. These PICs are assembled into SGs facilitated by the RNA chaperone Y box-binding protein-1 (YB-1) (33). (2) Inhibition of the interaction between eIF4A and eIF4G or inhibition of the RNA helicase activity of eIF4A is a mechanism by which most instances of SGs assembly are induced. 15-Deoxy-delta 12,14-prostaglandinJ2 (15d-PGJ2) is a natural lipid inflammation mediator belonging to the prostaglandin family that can bind eIF4A and thus prevent the interaction between eIF4A and eIF4G (34). Pateamine A (PatA) and hippuristanol disrupts the function of eIF4F by inhibiting the helicase, ATPase activity or RNA-binding ability of eIF4A (35). (3) Destruction of the eIF4G structure. For example, in coxsackievirus B (CVB) infection, the 2A protein cleaves eIF4G (36).

### 2.4 Factors inducing SGs formation

In the body, the amount of mRNA in a polysome is regulated mainly *via* the initiation of translation. When translation initiation is successful, mRNA is located within a polysome. When translation initiation is blocked, mRNA is in a free state and is diverted to form a SGs. Therefore, any factor that blocks translation initiation can promote SGs formation. Numerous stresses and agents can also induce SGs formation (37).

Low nutrient levels, hypoxia, and osmotic shock are endogenous stressors; arsenic compounds, diethyl maleate (DEM), and paraquat are environmental stressors; overexpressed amyloid-beta 42 (Aβ42), MG132 and lactacystin are proteasome inhibitors; dithiothreitol (DTT) and thapsigargin are ER stressors, carbonyl cyanide 4-(trifluoromethoxy) phenylhydrazone (FCCP) and clotrimazole are mitochondrial poisons, and sorbitol (38), among other compounds, promote SGs formation in a p-eIF2α-dependent manner. Furthermore, when combined with stress, puromycin (39) and salubrinal, translation-modulating compounds stimulate SGs production in a p-eIF2α-dependent manner.

Ultraviolet (UV) radiation and brain ischemia are environmental stressors, overexpression of G3BP1, TIA-1, TIAR, TTP, FMRP, cytoplasmic polyadenylation element-binding protein (CPEB), SMN, dual-specificity tyrosine-(Y)-phosphorylation-regulated kinase 3 (DYRK3), tRNA fragments, and sodium azide are mitochondrial poisons; pateamine A, hippuristanol, silvestrol, 15d-PGJ2, prostaglandin A1 (PGA1), selenite and other compounds promote SGs formation in a p-eIF2α-dependent manner.

For certain novel molecules, such as menadione, among environmental stressors, and salicylate and 6,7-dihydro-4-hydroxy-3-(2′-hydroxy[1,1′-biphenyl]-4-yl)-6-oxo-thieno[2,3-b]pyridine-5-carbonitrile (A769662), among other compounds, combine to promote SGs formation. However, whether these novel SGs inducers dependent on p-eIF2α remains to be determined. Heat shock, among environmental stressors, induces SGs production in a p-eIF2α-dependent manner in mammalian cells (40), but its effects are p-eIF2α independent in Drosophila, *C. elegans*, and yeast. H_2_O_2_ can inhibit or promote SGs assembly in a p-eIF2α-independent manner, depending on the cell species and H_2_O_2_ concentration. Cycloheximide and emetine, which are translation modulators, induce SGs formation in a p-eIF2α-independent manner, and ISRIB, a translation modulator, first promotes SGs assembly and then promotes SGs disassembly in a p-eIF2α-dependent manner.

In conclusion, SGs formation is closely related to the depolymerization of translation polysomes, and the increase in untranslated mRNA levels in cells is conducive to SGs assembly. The disassembly and homeostasis of SGs and polysomes are regulated *via* translation initiation mechanisms (12). Therefore, pharmacological interventions that disrupt homeostatic translation can exert a marked impact on SGs formation. For example, emetine traps mRNA in polysomes, promotes SGs component depolymerization and inhibits SGs assembly. Puromycin promotes ribosome detachment from translated mRNA and releases the cleaved mRNA from polysomes, which significantly increases SGs formation.

## 3 Biological properties of SGs

### 3.1 Dispelling traditional concepts of mRNA translation with respect to SGs

It was previously believed that the formation of SGs was accompanied by the stagnation of mRNA translation. However, the formation of SGs in cells induced by sodium arsenite treatment favored subsequent reovirus replication: sodium arsenite pretreated cells had higher levels of viral protein synthesis and viral titers. Meanwhile, viral core particles were located in SGs. Therefore, the formation of SGs did not inhibit the translation of reovirus mRNA in the early postinfection period (41). Whether it means that mRNA not only did not degrade in SGs, but would further be translated. Since scientists had not conducted in-depth studies on the mRNAs in SGs, it is unclear whether the translation mRNA sequestered into SGs is inhibited.

In 2020, Mateju et al. made a major discovery showing that SGs do not directly inhibit any step of mRNA translation and that the translation of mRNAs located in SGs is not blocked (42). Using live-cell single-molecule imaging, the authors of this study found that untranslated mRNAs were preferentially recruited to SGs and that mRNAs localized to SGs could be translated. The translational state of the mRNA that was translocated between the cytosol and SGs was unchanged. By quantifying the correlation between translation status and localization of SGs, the authors found that 30% of translated mRNAs colocalized with SGs signals, suggesting that SGs-associated transcripts are commonly translated. When the distribution of mRNAs in SGs and cytoplasm was evaluated according to their translation status, only a small fraction of translated (12%) and untranslated (19%) transcripts were found to be located in SGs, with most transcripts retained in the cytoplasm. In examining the relationship between mRNA translation status and mRNA location in SGs, most translated (76%) and untranslated (65%) mRNAs in SGs were found to be located within 0.3 μm of the detected SGs boundaries, with translated mRNAs showing a slight propensity to be located close to the surface (mean distance ± SD: 0.22 ± 0.16 μm vs. 0.27 ± 0.18 μm), but translated mRNA was also detected deep inside the SGs, in large SGs and 1 μm or farther from the SGs boundary. The distance of mRNA from the SGs border was independent of the intensity of the translated mRNA signal. These findings suggest that the position of the mRNA in SGs is not changed *via* translation (42).

### 3.2 The regulatory effects of SGs on innate immunity

Viral infection can activate innate immune signaling pathways. The innate immune system includes pattern recognition receptors (PRRs), which sense viral RNA and present it sequentially in the innate immune signaling pathway, eventually inducing the production of a large number of IFNs and inhibiting the replication and spread of the virus (43). Viral dsRNA activates PKR phosphorylation, which ultimately leads to inhibited mRNA translation, RNase L-mediated RNA degradation, etc. (44). In addition, viral nucleic acid molecules of RNA and DNA viruses activate the innate immune signaling pathway. The RNA and DNA produced during viral replication are mediated by cellular Toll-like receptors (TLRs), retinoic acid inducible gene-1 (RIG-1)-like receptors (RLRs), DNA-mediated IFN regulators (DAIs), activator of IFN (STING/MITA/ERIS/MPYS), DEAD box polypeptide 41 (DDX41) and cGMP/CAMP synthase (cGAS) (45). Among these nucleic acid recognition molecules, RLRs carry three RNA helicases, RIG-5, MDA5 and DHX58 (LGP2), which sense mainly RNA molecules in the cytoplasm. These RLRs distinguish viral RNA molecules from host cell RNA molecules. For example, RNA molecules synthesized by viruses usually carry a 5’-ppp modification at the 5’ end (5’-PPP-containing single stranded RNA), whereas RNA molecules synthesized by host cells do not carry this modification (46). In addition, RLRs also mediate the activation of innate immune signaling pathways. RIG-1 generally recognizes viral single-stranded RNA (ssRNA) and short dsRNA, and MDA5 generally recognizes long dsRNA produced during viral replication. After recognizing viral RNA, RIG-1 and MDA5 undergo a conformational change, bind to receptor proteins called virus-induced signaling adapters (VISAs), such as mitochondrial antiviral signaling protein (MAVS), IPS-1, and Cardif on the mitochondrial outer membrane, and subsequently recruit TRAFs and activate TBK1/IKKi and IKKa/IKKp complexes, which in turn activate IRFs and NF-κB; ultimately, activated IRF3, IRF7 and NF-κB enter the nucleus to activate the gene transcription and expression of IFN and inflammatory factors (47).

**Figure 3:**
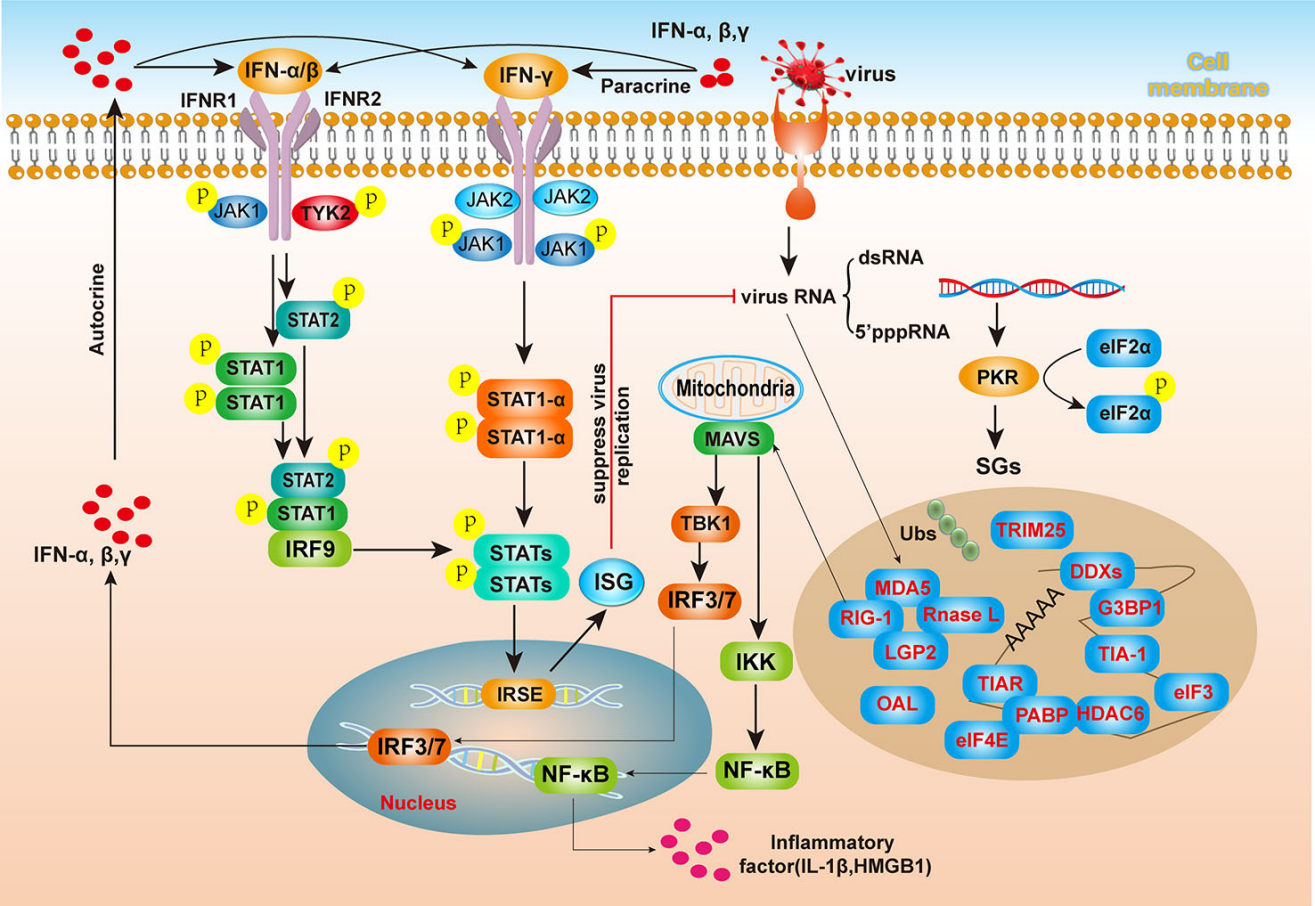
The relationship between SGs and antiviral innate immunity. After viral invasion into host cells, viral RNA (dsRNA or 5’pppRNA) activates the PKR kinase, resulting in eIF2α phosphorylation and SGs assembly. SGs contain the RIG-I-like receptors MDA5 and RIG-I, which can interact with MAVS on the mitochondrial membrane to activate the IFN signaling pathway and promote IFN production. After binding to IFN receptors on the cell membrane, interferon-stimulated genes (ISGs) induce the inhibition of viral RNA replication through the JAK/STAT pathway. After viral infection of cells, viral genomic RNA activates PKR, phosphorylates eIF2α, and inhibits the initiation of translation, thereby inhibiting viral protein synthesis and inducing SGs formation. SGs are composed of diverse components, including viral RNA, RNA-binding proteins (RBPs), and translation initiation factors, and especially, many innate immune pattern recognition molecules, such as MDA5, RIG-I, LDP2, and RNase L. These pattern recognition molecules bind viral RNA, which in turn transmits signals to MAVS on the outer mitochondrial membrane, activates TBK1/IKK phosphorylation, and allows transcription factors IRF3/7 and NF-κB to enter the nucleus, where they activate the expression and secretion of type I interferons and certain inflammatory factors. IFN activates intracellular STAT1/2 phosphorylation after autocrine or paracrine binding to cell membrane interferon receptors. Subsequently, p-STAT1/2 enters the nucleus together with IRF9, recognizes internal ribosome entry site (IRSE) regions, activates the expression of various ISGs, and produces antiviral effects.

SGs regulate innate immune responses. In recent years, certain sensors and elements involved in these signaling pathways, namely, RIG-1, MDA5, LGP2, and various sensors, such as PKR, RNase L, 2’-5’-oligoadenylate synthetase (OAS), have been found to be recruited to SGs, and multiple marker proteins of SGs have been found to be colocalized (48), suggesting a tight connection between SGs the formation and innate immune responses. Among the aforementioned sensors, OAS/RNase L cleaves viral RNA, and the cleaved viral RNA is recognized by pattern recognition molecules, which further activate the antiviral immune pathway. OASs are interferon-induced antiviral enzymes, and the human OAS family consists of four members: OAS1, OAS2, OAS3, and OAS-like protein (OASL). The profiles of SGs with assembly induced by viral infection and the innate immunity responses are shown in **Figure 3**. The interferon response protein molecule PKR recognizes viral dsRNA and 5’ppp RNA, senses the presence of viral nucleic acids in cells, and mediates downstream interferon responses. In addition, the activation of PKR induces the phosphorylation of eIF2α, which blocks the translational process in cells and leads to SGs formation (49). Cytoplasmic dsRNA and 5’ppp-RNA are also recognized by the pattern recognition molecules MDA5 and RIG-I, which are SGs components (50). After viral RNA is recognized by MDA5, RIG-I or LGP2, a signal is transmitted to MAVS (on the outer mitochondrial membrane. Subsequently, TBK1/IKK are phosphorylated, allowing the transcription factors IRF3/7 and NF-κB to enter the nucleus and activate the expression and secretion of type I interferons and certain inflammatory factors (51). In addition, after autocrine or paracrine IFN-α and IFN-β bind with IFN receptors on the cell membrane, leading to STAT1/2 phosphorylation in the cell. Phosphorylated STAT1/2 enters the nucleus together with IRF9, recognizes internal ribosome entry site (IRSE) regions, and activates the expression of more ISGs, producing antiviral effects.

Notably, many proteins encoded by ISGs that induce IFN signaling pathway activation are recruited to SGs and regulate SGs formation, and SGs also create platforms for a host antiviral response (52). OASs induced by IFN recognize dsRNA and then catalyze the production of 2-5A from ATP, thereby inducing dsRNA degradation by RNase L, an SGs component. ADAR1, another SGs protein, is also activated by IFN to convert A to I in dsRNA, thereby altering viral dsRNA and interfering with viral replication (53). SGs formation induced by which virus is not a result of IFN signaling pathway activation, but when G3BP1 is silenced, the ability of influenza A virus (IAV) infection to induce SGs formation in cells is significantly weakened, and the IFN activity level is also significantly reduced (54).

Specific viruses exhibit unique regulatory programs. For example, the viral protein NS1 in IAV strongly inhibits SGs formation initially induced by IAV and activates innate immunity mediated by RIG-1. Proteins that engage in signaling mediated through IFN receptor 1 (IFNAR1) and are encoded by ISGs establish an antiviral state in both infected and uninfected cells (55). In addition to the aforementioned molecular mechanisms, PKR activates inflammasome and macrophage responses during bacterial and dsRNA virus infection, resulting in the release of the cytokines IL-1β and HMGB1 (56).

In summary, virus-induced SGs formation generates intracellular platforms through which pattern recognition molecules regulate downstream signaling (57). The specific regulatory mechanisms are relatively complex, and research in recent years has revealed that interactions, such as the interaction between OASL and MDA5, enhance MDA5-mediated type I interferon signaling pathway activation (58). TRIM25 can also be recruited to SGs and is required for full RIG-I activation (59). Two types of enzymes are encoded by ISGs: one type constitutes dsRNA-binding proteins, such as the protein kinase PKR, which is regulated by dsRNA, and the other type comprises adenosine deaminase ADAR1, which acts on dsRNA (60). G3BP1 contributes to the induction of antiviral cytokine IFN-β production induced by RIG-1 (61). Studies are gradually revealing the connections among various innate immune signaling pathways in cells, SGs and various functional interactions.

## 4 SGs and viruses

### 4.1 Viruses and SGs coexist

After a virus infects a cell, it causes a series of changes in the intracellular environment, and after these changes are sensed by cells, the series of stress responses are initiated to resist viral infection and promote cell survival. However, a virus may evade or even exploit the stress responses of host cells in a number of ways to promote their own replication. SGs formation is a stress response in cells that can inhibit translation; however, viruses can regulate and change SGs assembly in various ways. Therefore, the relationships between viruses and SGs are diverse. Generally, the relationships can be roughly classified into three categories: viral infection is induced first and then SGs formation is inhibited; viral infection inhibits SGs formation; and viruses and SGs coexist. Therefore, as shown in **Table 1**, various modes of viral infection of host cells affects SGs: After viral infections, SGs are quickly disassembled after formation, stable SGs are formed, or SGs are not formed. After the initial synthesis of viral proteins, positive-strand RNA viruses switch individual genome recruitment of ribosomes to translational repression to clear ribosomes from templates and facilitate viral RNA replication (114). Therefore, a large number of RNA regulatory proteins are involved in viral replication. The key SGs protein G3BP1 interacts with nonstructural proteins (NSPs) of the virus to form complexes that inhibit viral replication. Studies have shown that G3BP1 forms a complex with the viral polymerases nsp3, nsp2 and nsp4 (115).

**Table 1:** Viral infection and SGs formation.

Classification	Virus	SGs formation	Mechanism of induction or inhabitation	Ref
*Picornaviridae*	PV	Y(T)	3C protein cleaves G3BP1	(62, 63)
	EMCV	Y(T)	3C protein cleaves G3BP1, Leader protein inhibits SGs	(64, 65)
	CV	Y(T)	3C protein cleaves G3BP1	(66)
	MV	N	Leader protein inhibits SGs	(64)
	TMEV	N	Leader protein inhibits SGs	(64)
	SAFV	N	Leader protein inhibits SGs	(64)
*Adenoviridae*	AdV5	Y(T)	E1A mediates several rounds of SGs assembly and disassembly	(65)
*Reoviridae*	MRV	Y(T)	SGs induction by MRV requires viral uncoating	(67, 68)
	RV	Y(T)	Induces SGs formation and sequestration	(69)
*Togaviridae*	SINV	Y(T)	ATG16L1 induces SGs formation, and ZAP recruits SGs	(65, 70, 71)
	SFV	Y(T)	nsp3 traps G3BPs into viral granules, and viral translation enhancers inhibit SGs formation	(72–74)
	CHIKV	N	nsp3 recruits G3BP1 to repress SGs formation through the SH3 domain-binding motif	(75)
	RUBV	Y	The mechanism of SGs formation induced by RUBV is unclear.	(76)
*Orthomyxoviridae*	IAV	N	NS1 blocks the PKR pathway *via* RNA sequestration; NP inhibits SGs formation; PA-X promotes poly(A) RNA-binding protein to the nucleus to inhibit SGs formation	(54, 77, 78)
*Paramyxoviridae*	MeV	N	Protein C inhibits PKR by activating ADAR1	(17)
	RSV	Y	PKR-dependent induction of SGs, the 5’-end of the genome trailer domain represses SGs formation	(79–81)
	NDV	Y	Induces the formation of antiviral SGs	(82)
	SeV	N	Protein C and protein V inhibit PKR activation, and trailer RNA interacts with TIAR to inhibit SGs formation	(83, 84)
*Flaviviridae*	HCV	Y(T)	Activation of PKR in the 5’ untranslated region of the genome induces SGs formation and GADD34, ATX2, and NS5B mediate the repression of SGs formation	(85–89)
	WNV	N	Genomic 3’-UTR binds and deactivates TIA-1 and TIAR suppresses SGs formation	(90)
	DENV	N	Genomic 3’-UTR binds and deactivates TIA-1 and TIAR, inhibits p38-Mnk1 signaling and eIF4E phosphorylation, and inhibits SGs formation	(90, 91)
	ZIKV	N	Hijacks G3BP1, TIAR and caprin-1, inhibits eIF2α phosphorylation, inhibits SGs	(92, 93)
	JEV	N	Core protein interacts with caprin1 to inhibit SGs formation	(94)
*Retroviridae*	HIV	N	Gag interacts with G3BP1 and eEF2, and then downregulates phosphorylated 4EBP1 to inhibit SGs formation	(95, 96)
	HTLV-1	N	Tax protein interacts with HDAC6 to inhibit SGs formation	(97)
*Herpesviridae*	HSV-1	N	VHS and US11 block PKR activation, and UL41 mutation causes SGs accumulation	(98–102)
	HSV-2	N	VHS inhibits SGs formation	(100)
	HCMV	N	pTRS1 and pIRS1 antagonize PKR to inhibit SGs formation	(103–105)
	KSHV	N	RNA-binding motifs of ORF57 regulate PKR repression of SGs formation	(106, 107)
*Poxviridae*	VACV	Y	Induces SGs formation, and E3 protein deletion induces AVG formation	(108–110)
*Coronaviridae*	CoV	Y	PTB colocalizes with TIA-1 and TIAR to promote SGs formation	(111)
*Rhabdoviridae*	VSV	Y	Induces SGs-like particle formation	(112)
*Arenaviridae*	JUNV	N	Nucleoprotein or GPC inhibits SGs formation by inhibiting eIF2α phosphorylation	(113)

4EBP1, eukaryotic translation initiation factor 4E-binding protein 1; ADAR1, adenosine deaminase acting on RNA1; ATG16L1, autophagy-related protein 16-like 1; ATX2, ataxin-2; AVGs, antiviral granules; Caprin-1, cytoplasmic activation/proliferation-associated protein-1; CV, Coxsackievirus; DENV, Dengue virus; eEF2, eukaryotic elongation factor 2; eIF2α, eukaryotic translation initiation factor 2α; eIF4E, eukaryotic initiation factor 4E; EMCV, encephalomyocarditis virus; GADD34, growth arrest and DNA damage-inducible transcript 34; GPC, glycoprotein precursor; HCMV, human cytomegalovirus; HCV, hepatitis C virus; HDAC6, histone deacetylase 6; HIV, human immunodeficiency virus; HSV-1, herpes simplex virus type-1; HSV-2, herpes simplex virus type-2; HTLV-1, human T-cell leukemia virus type-1; IAV, Influenza A virus; JEV, Japanese encephalitis virus; JUNV, Junin virus; KSHV, Kaposi’s sarcoma-associated herpesvirus; MeV, measles virus; MV, mengovirus; Mnk1, mitogen-activated protein (MAP) kinase signal-integrating kinase 1; NP, nucleoprotein; NS1, nonstructural protein 1; NS5B, nonstructural protein 5B; nsp3, nonstructural protein 3; ORF57, open reading frame 57; PA-X, polymerase-acidic protein-X; PKR, protein kinase R; PTB, polypyrimidine tract-binding protein; PV, poliovirus; RV, rotavirus SAFV, scaffold virus. MRV. mammalian orthoreovirus; SeV, Sendai virus; SGs, stress granules; TIA-1, T-cell-restricted intracellular antigen 1; TIAR, TIA-related protein; TMEV, Theiler’s murine encephalomyelitis virus; VACV, vaccinia virus; CoV, coronavirus; VHS, virion host shutoff; VSV, vesicular stomatitis virus; WNV, West Nile virus; ZAP, zinc-finger antiviral protein; ZIKV, Zika virus; N, no; T, transient; Y, yes; Ref, references.

#### 4.1.1 Viral infection induces and then suppresses SGs formation or leads to SGs disassembly

Viruses, especially RNA viruses can cause the formation of SGs-like bodies in the cytoplasm. Some viruses induce SGs formation by activating the eIF2α kinases PKR and GCN2. When cells detect viral RNA in the cytoplasm, these two factors are activated and play specific roles. Viral infection produces a unique type of cellular stress response and often induces the formation of viral-type SGs (V-SGs), and some V-SGs specifically contain the RBP SRC associated in mitosis of 68 kd (Sam68) (116). As shown in **Table 1**, during infection with viruses of the *Picornavirus family*, such as poliovirus (PV) (63), CVs (66), encephalomyocarditis virus (EMCV) (65), and Theiler’s murine encephalomyelitis virus (TMEV) (64), SGs formation is transient and closely related to the viral protease 3C. However, in the later stages of infection, the breakdown of the G3BP1 or L protein in the cell leads to the disintegration of SGs and inhibits the formation of SGs (62–64). The *Flaviviridae* family member Hepatitis C virus (HCV) induces the formation of SGs which are dissembled in later infection stages (85, 88, 89) (86). The *Adenoviridae* family member Adenovirus type 5 (AdV5) can induce several rounds of SGs formation and degradation (65). The *Reoviridae* family member Mammalian orthoreovirus (MRV) induces the SGs formed in the early stage, this in turn promote the replication of the virus (67) (117–119). Rotavirus (RV) infection can lead to eIF2α phosphorylation but inhibit SGs formation. However, RV can induce SGs formation and SGs sequestration by viroplasms (VMs) to promote viral replication in early stage (69).

The *Togaviridae* family member Sindbis virus (SINV) induces SGs formation in the early stages of infection, a process requiring autophagy-related protein 16-like 1 (ATG16L1), but then, the SGs disappear in the late stages of infection (65, 70). Rubella virus (RUBV) infection promotes SGs assembly, but the number of SGs decreases in the late stages of infection, and the specific molecular mechanisms involved in these changes are unknown (76). Replication of Semliki forest virus (SFV) RNA induces SGs formation that depends on the level of eIF2α phosphorylation. Throughout viral translation, viral nonstructural protein 3 (nsp3) traps G3BP1 into viral granules, thereby inhibiting the formation of SGs and promoting viral replication (74). In addition, the viral translation enhancer near the initiation codon of SFV viral RNA, AUG promotes SG disassembly during infection (73).

#### 4.1.2 Viral infections that inhibit SGs formation

During the life cycle of many viruses, SGs formation is not observed in cells. In some cases, SGs failed to form despite high p-eIF2α levels in infected cells or exposure to new SGs-induced stress. These outcomes indicate that some viral infections inhibit SGs formation. As shown in **Table 1**, the *Picornaviridae* family Mengovirus (MV), EMCV, TMEV and Saffold virus (SAFV) members inhibit the formation of SGs through L protein and interferon (IFN) (64). The *Retroviridae* family members human immunodeficiency virus (HIV) and human T-cell leukemia virus type-1 (HTLV-1) inhibit SGs formation through the Gag protein and Tax protein (95–97). The *Paramyxoviridae* family member Measles virus (MeV) and Sendai virus (SeV) inhibit SGs formation through protein C and protein V (17, 83, 84). The *Orthomyxoviridae* family member IAV inhibit SGs formation through NS1 protein, nucleoprotein (NP) (54, 78, 120). The *Flavivirus* family members West Nile virus (WNV) (121), Dengue virus (DENV) (91), ZIKA virus (ZIKV) (93) and Japanese encephalitis virus (JEV) (122) inhibit SGs assembly by PKR, genomic 3′-UTR, p38/mitogen-activated protein (MAP) kinase signal-integrating kinase 1 (Mnk1) signaling, or Caprin1 (90) (91–94, 123). The *Herpesviridae* family member herpes simplex virus type-1 (HSV-1), herpes simplex virus type-2 (HSV-2), Human cytomegalovirus (HCMV), Kaposi’s sarcoma-associated herpesvirus (KSHV), Pseudorabies virus (PRV) inhibit SGs formation through virion host shutoff (VHS) protein, US11 protein, unfolded protein response (UPR), p-TRS1 and p-IRS1 proteins, open reading frame 57 (ORF57), growth arrest and DNA damage-inducible protein 34 (GADD34), protein phosphatase 1 (PP1) (98, 100–102, 104, 105, 107, 124, 125). The *Togaviridae* family member Chikungunya virus (CHIKV) inhibits SGs formation by Nsp3 (75). The *Arenaviridae* family member Junin virus (JUNV) inhibits SGs formation through the nucleoprotein (N) or glycoprotein precursor (GPC) (113).

#### 4.1.3 The coexistence of viral infection and SGs

As shown in **Table 1**, the *Poxviridae* family is a group of enveloped dsDNA viruses that cause smallpox, monkeypox, and vaccinia in humans. Most research on poxviridae and SGs has focused on vaccinia virus (VACV). VACV leverages different SGs proteins at different replication stages to facilitate the completion of each replication cycle (126). Some VACV mutant-infected cells form SGs containing components such as TIA-1, and these structures are called antiviral stress granules (avSGs) due to their ability to inhibit viral replication (108–110). It is worth noting that avSGs is a type of SGs. The avSGs are not formed in all virus-infected cells. Although some cells infected with the virus form SGs, these SGs do not have antiviral ability and instead disappear in the later stages of infection. The presence of SGs facilitates both viral proliferation and host growth. The aforementioned studies have shown that SGs and VACV coexist, and the components of SGs can promote the transcription and translation of VACV. The *Paramyxoviridae* family member respiratory syncytial virus (RSV) can induce SGs formation in some cells after infection and can form large inclusion bodies in cells with SGs formation depending on the PKR-mediated eIF2α phosphorylation pathway (81). The SGs formation may facilitate the translation and replication of this virus by inhibiting the translation of host cells (80). However, only RSV with mutations in the 5’-end of the genome have been reported to induce SGs formation, even though the 5’-end of the viral genome is required for the transcription of RSV (79). Newcastle disease virus (NDV), another *Paramyxoviridae* virus, can form antiviral SGs similar to those induced by VACV in infected cells. When the formation of antiviral SGs was inhibited, NDV-induced interferon levels were greatly reduced (82). The *Coronaviridae* family menber coronavirus (CoV) colocalizes TIA-1 and TIAR with polypyrimidine tract-binding protein (PTB) to promote SGs formation (111). The *Rhabdoviridae* family member vesicular stomatitis virus (VSV) induces the production of particles that contain PCBP2, TIA1, and TIA1 proteins. These particles are exclusively located in SGs-like structures (112).

### 4.2 SGs inducers are expected to become novel antiviral drugs

Drug-induced PKR activation of SGs formation generated by p-elF2α is thought to control viral infection. TIA-1 and its homolog TIAR constitute a class of proteins that specifically bind to the 3’-non-coding region of TNF-α and matrix metalloproteinase-13 (MMP-13) mRNA and exert a translational silencing effect. Both proteins carry three RNA recognition motifs that bind with high affinity to U- and A+U-rich RNAs. Sustained TIA1 or TIAR expression inhibits cell proliferation, favors cell cycle arrest in the G1/S phase, and triggers cell death through slow caspase-dependent apoptosis and late autophagy (127). Therefore, viral spread can be inhibited by inducing the aggregation of TIA-1 (127). *In vitro* studies have shown that eIF4A helicase inhibitors exert antiviral effects, pateamine A inhibits replication of IAV and cytomegalovirus (128), and silvestrol effectively inhibits Ebola virus (EBOV) replication (129). eIF4A helicase inhibitors have been shown to exhibit antiviral activity at concentrations that do not lead to significant cytotoxicity. Therefore, it is of great value to develop relevant antiviral agents for determining the interaction of different viruses with SGs.

## 5 SGs and bacteria and other microorganisms

Some scholars have proposed that bacteria affect the level of p-eIF2α and maintain a close relationship with SGs (9). Gram-negative bacteria such as Shiga toxin-producing *Escherichia coli* (STEC) (130), *Aeromonas hydrophila* (131), *Vibrio vulnificus* (*V. vulnificus*) (132), *Pseudomonas aeruginosa* (PA) (133), and *Yersinia pseudotuberculosis* (134) significantly increased p-eIF2α levels in cell lines *in vitro*. In a study of the formation and kinetics of SGs in bacteria, it the ability of the SGs markers eIF3 and eIF4B to move to SGs was found to be inhibited in *Shigella flexneri*-infected cells, and the aggregation of SGs containing G3BP1 and eIF4G was also inhibited. Further studies have shown that invasive bacteria exhibit this inhibitory effect based on conditions that promote eIF2α phosphorylation or inhibit eIF4A helicase activity (135). The assembly of SGs requires the involvement of dyneins, which that regulate microtubule movement. During the inhibition of SGs formation mediated by *S. flexneri*, the acetylation level of alpha-tubulin was significantly increased, and the number of alpha-tubulin-mediated microtubule bundles, microtubule stability, and microtubule-based transport and regulatory functions were subsequently affected (135).

Gram-positive bacteria and Gram-negative bacteria induce elevated p-eIF2α levels. However, the species found to cause elevated p-eIF2α levels were members of the Firmicutes phylum, namely, Bacilli (*Listeria monocytogenes*, *Streptococcus pneumoniae*, and *Staphylococcus aureus*) or Clostridia (*Clostridium difficile*) (9). *Listeria monocytogenes*, one of the gram-positive bacteria, was the most studied p-eIF2α-inducing species (9). Mycobacteria belong to the gram-positive Actinobacteria phylum and are characterized by their “acid resistance”, which prevents gram-positive staining due to lipids attached to the cell wall. However, the ability of other mycobacteria to induce p-eIF2α in host cells appeared to be no different. Notably, *Mycobacterium avium* (136), *Mycobacterium ulcerans* (137), and *Mycobacterium tuberculosis* (138, 139) can activate the PERK/eIF2α/CHOP pathway.

In addition, other pathogens, such as *Mycoplasma hyopneumoniae*, can activate the PERK/eIF2α/CHOP pathway (140). Studies have reported that, three protozoan species, *Leishmania amazonensis* (141), *Plasmodium berghei* (142), and *Toxoplasma gondii* (143), and one fungal species, *Histoplasma capsulatum* (144), can increase p-eIF2α levels, after an organism infects the cell, the host p-eIF2α level increases. However, for some microorganisms, the change in p-eIF2α level does not involve a constant increase with the change in infection time, and therefore, more research in the future is needed to confirm the patterns of p-eIF2α level changes and microorganism infection (9).

## 6 SGs and inflammation

Similar to their relationship after viral infection, SGs and inflammatory factors can interact. Studies on the effect of SGs on inflammation have reported that in primary bone marrow-derived macrophages stimulated by endotoxin, SGs can reduce the degree of cellular inflammatory damage (10). SGs can also inhibit apoptosis by reducing the production of reactive oxygen species (145). An important component of SGs, DDX3X, competitively binds to NLRP3, thereby inhibiting inflammasome activation and regulating the “survival-related pyroptosis” of cells (10). The core protein HuR in SGs can bind to the 3′-UTR of COX-2 to stabilize its mRNA (146).

In contrast, certain inflammatory factors are directly or indirectly related to SGs formation. In mucosal inflammation, the proinflammatory factors IFN-γ and TNF-α cause the phosphorylation of eIF2α to induce SGs formation, and HSP70 mRNA is encapsulated into SGs, reducing the expression of HSP70 (11). SGs generated after heat shock can recruit TRAF2 and inhibit TNF-α-mediated activation of NF-κB through its interaction with eIF4G (147). In the immune response, eIF2α in T cells is phosphorylated after the first antigen is presented to form SGs, which encapsulate cytokine mRNA. When the antigen is re-presented by T cells, a mechanism of SGs disassembly is initiated, and mRNA is released for the translation and secretion of cytokines (148). Atherosclerotic inflammation promotes the formation of SGs that accumulate in endometrial macrophages and vascular smooth muscle cells (VSMCs) as the disease progresses, sequester mRNA transcripts and halt translation. Knocking down G3BP1 in VSMCs inhibits SGs formation while altering the inflammatory gene expression profile (149), and IL-19 can reduce vascular inflammation by reducing the rate of SGs formation and alleviate atherosclerosis (149).

Currently, the impact of SGs on the inflammatory response has not been determined. Different diseases exhibit different anti-inflammatory or proinflammatory effects of SGs or their key components. In the process of atherosclerosis, SGs can be formed in vascular smooth muscle cells (VSMCs). Decreasing the expression of G3BP2, a key component of SGs, can reduce oxidized low-density lipoprotein (ox-LDL) - induced inflammation in the atherosclerotic lesions. This study only explored the effect of G3BP2 on the atherosclerotic lesions (150). However, it remains unclear whether inhibiting the expression of G3BP2 leads to a decrease in the formation of SGs, and the subsequent reduction in vascular inflammation levels during the process of atherosclerotic lesions. The anti-inflammatory drug cortisone can induce the production of SGs (151). Whether the powerful anti-inflammatory effects of cortisone are mediated by SGs will be a very interesting issue. The signaling -lipid molecule 15-deoxy-delta 12,14-prostaglandin J2 (15d-PGJ2) inhibits translation leading to an increase in SGs production and TRAF2 is sequestered in these SGs. The sequestration of TRAF2 contributes to the anti-inflammatory activity of 15d-PGJ2 (34). Carbon monoxide (CO) can play an anti-inflammatory role by inducing the formation of SGs (152). SGs could protect hepatocytes from hypoxia-induced damage during acute liver failure by reducing endoplasmic reticulum stress (ERS)-mediated apoptosis (153). ERS is a key process in mediating inflammatory responses, so molecules related to the ERS pathway can serve as potential anti-inflammatory targets for SGs.

## 7 Discussion

Cells are often exposed to potentially fluctuating adverse environmental conditions, and SGs formation enables cells to adapt to different environmental changes as SGs provide protection for important cellular components. SGs can transduce signals in response to cellular stress and show protective functions. The formation and function of SGs are tightly regulated dynamic processes. When the translation initiation mechanism is blocked, mRNA and many translation-related proteins aggregate into SGs and are temporarily stored in SGs to prevent the mediation of degradation mechanisms. Moreover, mRNAs can also be translocated into SGs from P granules or cytoplasmic mRNAs. During an integrated stress response, cells trigger phosphorylation of the TIF eIF2α, resulting in the inhibited translation of most mRNAs. This inhibitory state allows cells to conserve energy and use their resources to restore homeostasis. During stress, SGs temporarily store mRNAs and proteins and protect both types of molecules from autophagy and proteasome degradation, with cells rapidly restarting translation and activating other signaling pathways after recovery from stress. The aggregation and depolymerization in SGs partly depend on phase separation, interactions between microtubules and dyneins, and RBPs.

In addition to protecting mRNA from degradation, promoting mRNA transcription in SGs, and regulating innate immunity, SGs have also been implicated in apoptosis and other intranuclear processes (154). In addition, the key molecules formed by SGs can interact with some molecules in pathogens. Based on the aforementioned molecular mechanisms, SGs have been shown to be involved in viral infections; bacterial infections; novel pathogen infections; inflammatory diseases; cancers; various neurological diseases, including Fragile X syndrome, spinal muscular atrophy, spinocerebellar ataxia, myotonia; and pathological processes such as malnutrition responses. However, the functions of SGs differ depending on the virus, bacteria, inflammatory response and host cell involvement (80, 155).

Recently, research on SGs has been focused mainly on infectious diseases, especially viral infections. Three modes of viral infection and SGs formation have been described. Some viruses induce SGs production in the early stage of infection. However, most viruses inhibit SGs formation during a certain period in the infection cycle. When the viral gene expression level is high, SGs coexist in virus-infected cells, and some viruses leverage SGs to facilitate their own replication. In the process of virus infection, SGs formation is induced in various ways. Among the SGs formation processes, dsRNA or RNA with novel structures produced *via* virus replication is recognized by PKR, which is the most common way in which viruses induce SGs formation. The shutdown of host cell translation by viral components is another mechanism by which viruses induce SGs formation. SGs are the products of the mutual gamesmanship observed between viruses and cells. Viral infection of cells can cause eIF2α phosphorylation, but p-eIF2α does not necessarily lead to SGs formation. SGs not only directly inhibit viral translation but also promote cells survival by inducing innate immunity to suppress viral replication. For example, SGs formed by RSV infection show enhanced replication, while SGs formed by MRV infection show inhibited replication. The SGs that inhibit viral replication are attacked by viruses at a later stage of formation, resulting in no prolonged assembly or even the disassembly of SGs. The constituent proteins in SGs also exert effects on viral replication, and the replication efficiency of MRV is significantly increased in G3BP1-knockout cells. In conclusion, when host cells are infected by viruses, they protect themselves by regulating SGs formation, and a virus also targets SGs to benefit itself. Therefore, the interactions between different viruses and different cells are not the same, and the mechanisms underlying these interactions may also be dynamic developmental processes.

Compared with that of RNA viruses, the regulatory mechanism of DNA virus on SGs formation has been less frequently reported. Whether SGs formation contributes to the antiviral effects of these compounds is unclear, and inhibition of viral protein synthesis is likely sufficient to disrupt viral replication. The speculation that SGs compete with bacteria and other pathogens has been based on a large number of related studies of SGs and viruses, which have provided great references for future research directions. We believe that in the future, more studies on SGs related to bacterial infection and the inflammatory response will be reported, and some valuable targets for antiviral, antibacterial, and anti-inflammatory treatment will be found.

However, many limitations in the research on SGs are evident. Under different stimuli, the components of SGs differ, and the specific composition of SGs is not fully understood. With in-depth related research, some novel proteins in SGs have been gradually identified. Recently, many reports on the interaction between SGs and various viruses have become available, but the specific molecular mechanisms are still unclear, and detailed research is lacking. Because SGs lack membranes, SGs components cannot be directly extracted or identified, and SGs are aggregates with dynamic formation and disaggregation kinetics, which makes studying their nature difficult. No effective method to detect SGs in tissues has been established; therefore, the study of SGs has remained at the cellular level, which has resulted in an in-depth study of the relationship between SGs and diseases. Therefore, drugs that activate p-elF2α through PKR to promote SGs formation are expected to be a developed to control viral infection, but it is unclear whether SGs formation contributes to the antiviral effects of these compounds, and they likely exert an inhibitory effect on viral protein synthesis. Whether SGs can be targeted in effective strategies for inducing bacteriostasis or anti-inflammation effects or inhibiting tumor growth remains unknown due a lack of relevant research.

However, with increasingly in-depth research, especially in recent years, it has been revealed that the translation of mRNA in SGs is not suspended but is independent of the type of cell and is mediated by a complete set of transcriptional and translational regulatory systems. Many problems identified in previous studies, such as how polyadenylation and the RNA-induced silencing complex (RISC) affect the relationship between SGs and mRNA and how viral RNA escapes from SGs and is translated in the presence of high concentrations of phosphorylated eIF2α, have been partially resolved. Moreover, translationally blocked mRNA promotes SGs formation, and with an increase in the mRNA levels, the number of SGs formed is increased. SGs contain mRNAs that are protected from degradation and for which translation has been initially blocked, but these mRNAs can be replicated in SGs. Moreover, in-depth study into SGs and infectious and inflammatory diseases will help to identify potential therapeutic targets for diseases.

## Author contributions

WL and YW wrote the manuscript, completed the review process, contributed to the conception and design of manuscript. YW contributed to the conception and design of the study. All authors contributed to the article and approved the submitted version.
